# Protective eyewear in children with one eye vision loss: compliance and trends

**DOI:** 10.1007/s00417-024-06720-6

**Published:** 2024-12-23

**Authors:** Tal Yahalomi, Daphna Mezad-Koursh, Amir Sternfeld, Miriam Ehrenberg, Anat Bachar Zipori, Gad Dotan

**Affiliations:** 1 Department of Ophthalmology, Samson Assuta Ashdod Hospital, Ashdod, Israel; 2 The Faculty of Health Sciences, Ben-Gurion University of the Negev, Beersheba, Israel; 3 Division of Ophthalmology, Tel-Aviv Sourasky Medical Center, Tel-Aviv, Israel; 4 Tel-Aviv Faculty of Medical and Health Sciences, Tel-Aviv University, Tel-Aviv, Israel; 5 Ophthalmology Unit, Schneider Childrens Medical Center of Israel, 14 Kaplan Street, PO Box 559, 4920235 Petah Tikva, Israel

**Keywords:** Protective glasses, Protective eyewear, One-eye vision loss

## Abstract

**Purpose:**

Safety glasses are an important measure to prevent blindness, especially in one- eyed patients. However, patient compliance with eye protection is often limited. Unlike previous studies that described protective eyewear wearing in anophthalmic patients, this study analyzed their usage in functionally one-eyed children, having a significantly reduced visual acuity in one eye, determining common obstacles to their use.

**Methods:**

A survey-based study analyzing protective eyewear usage in children with one eye vision loss (mean logarithm of the Minimum Angle of Resolution (logMAR) visual acuity ≤ 0.7).

**Results:**

This study included 83 functionally one-eyed children (44 males), who received a recommendation to wear safety glasses. Ninety-nine percent of their caregivers were aware of this recommendation; however, 31% of them did not know the glasses’ true purpose. Regarding actual usage, only 29 (35%) children wore safety glasses at least 90% of the day, 26 (31%) children wore them part-time (10–90% of the day) and 28 (34%) wore them rarely or never at all (< 10% of the day). Compliance was higher when glasses provided optical correction. Reasons provided for non-compliance included: discomfort, appearance, cost and vision reduction. Many respondents recollected incidents in which the glasses prevented an eye injury, and less commonly an eye injury occurring while the child was not wearing eye protection.

**Conclusions:**

Compliance with safety glasses in functionally one-eyed children is limited. Emphasizing that numerous gaps and barriers need to be bridged to improve eyewear protection in these children to prevent trauma in the better-seeing eye with its devastating lifestyle effect.

****Key messages**:**

*****What is known***:**

- Anophthalmic patients often do not wear protective glasses, which are needed to prevent trauma to their only remaining eye.

*****What is new***:**

- Non-anophthalmic children with reduced visual acuity in one eye use protective glasses even less often, even when doing sports.
- Common reasons for not wearing safety glasses include: discomfort, dislike of self-appearance with glasses, and lens-induced vision reduction.

## Introduction

It is estimated that 2.4 million eye injuries occur annually in the United States, with nearly 35% of injuries among persons under 17 [1], most commonly during sporting or recreational activities [2]. Approximately 8.9/100,000 of these injuries are severe enough to require hospitalization [1]. Sports equipment, stones, wooden sticks, BB guns, slingshots, can lids, snowballs, and toys are the items most often implicated [3]. The American Academy of Ophthalmology estimates that 90% of these injuries would have been prevented if protective eyewear measures were worn at the time of trauma [4].

Although vision loss in one eye is not generally defined as a visual impairment, it harbors an increased risk of trauma to the unimpaired eye, which can cause permanent blindness and a permanent change in lifestyle [5]. Many activities such as ambulation, driving and learning that can easily be performed with only one seeing eye may become impossible in case vision in the other eye is also damaged. To prevent this devastating occurrence, ophthalmologists typically prescribe safety glasses to patients with one seeing eye to protect their other eye. However, it can be assumed that some of these patients will not wear the glasses as recommended, especially when the remaining eye is nearly emmetropic.

Previous studies assessing the employment of protective eyewear in one-eyed individuals included only anophthalmic patients, or mixed populations of children and adults [6, 7]. The purpose of this study is to evaluate compliance with protective eyewear in functionally one-eyed children, who have a significantly reduced visual acuity in one eye (mean logarithm of the Minimum Angle of Resolution (logMAR) visual acuity ≤ 0.7) but who are not anophthalmic, which is a more prevalent clinical scenario, assessing common obstacles to their use.

## Methods

We conducted a survey-based study of all children (< 18 years old) with functional vision loss in one eye, who received a recommendation to wear protective glasses from 2010–2022 at two Medical Centers to evaluate compliance with safety eyewear and identify common barriers to protective spectacle usage. The study received approval from both institutions’ research committees and was conducted following the rules and regulations of the Helsinki Declaration. A telephone informed consent was obtained from all participants. All children had a logMAR visual acuity of ≤ 0.7 in one eye and ≥ 0.3 in the other eye. Children with less than 6 months follow-up were excluded.

The following data were collected in all participants: date of birth, gender, cause of vision loss, date when initial recommendation for protective glasses was made, visual acuity in both eyes, cycloplegic refraction in both eyes, and spectacles prescription. Glasses having an optical correction of myopia ≥ −0.50 diopters, hyperopia ≥  + 2.00, or astigmatism ≥ 1.50 diopters in the better-seeing eye were defined as providing significant optical correction. Four authors (TY, ME, AS, GD) conducted the telephone survey with caregivers of patients who met the inclusion criteria (Appendix). The survey included questions about safety glasses usage, children’s and caregivers’ understanding of their glasses’ importance and possible compliance barriers limiting their usage.

Statistical analysis was performed using Prism 7 statistical software (GraphPad Software Inc., San Diego, CA). Categorical variables were summarized as frequencies and percentages. Continuous variables distribution was evaluated using histograms and the Shapiro Wilk test and were described using mean and standard deviation or as median and interquartile range. Fisher's exact test was used to compare categorical variables. All statistical tests were two-tailed and statistical significance was defined at a *p* level < 0.05.

## Results

Overall, we identified 94 children who were recommended to wear protective glasses during the study period, and 83 of their caregivers (88%) agreed to participate in the telephone survey and were thus included in our final analysis. Mean age of children (44 males, 53%) when protective eyewear was prescribed was 5.2 ± 4.1 years, and at the time of interviewing, it was 9.7 ± 5.1 years.

Thirty-two children (39%) had vision loss in one of their eyes at birth or soon after from congenital causes including congenital cataract, persistent fetal vasculature, optic disc or foveal hypoplasia, and choroidal coloboma, whereas 51 children (61%) had vision loss at an older age secondary to acquired conditions including refractive amblyopia, ocular trauma, brain tumors, retinal detachment, and exudative retinopathy.

At the time when protective eyewear was first recommended, visual acuity in healthy eyes was always better than in eyes with diminished vision and hence no significance test was performed (Mean logMAR visual acuity 0.13 ± 0.21 vs. 1.90 ± 0.88). Twenty-five (30%) were prescribed glasses providing an optically significant correction in the “good” eye, whereas the remaining 58 (70%) received glasses for protective purposes alone.

**Fig. 1:**
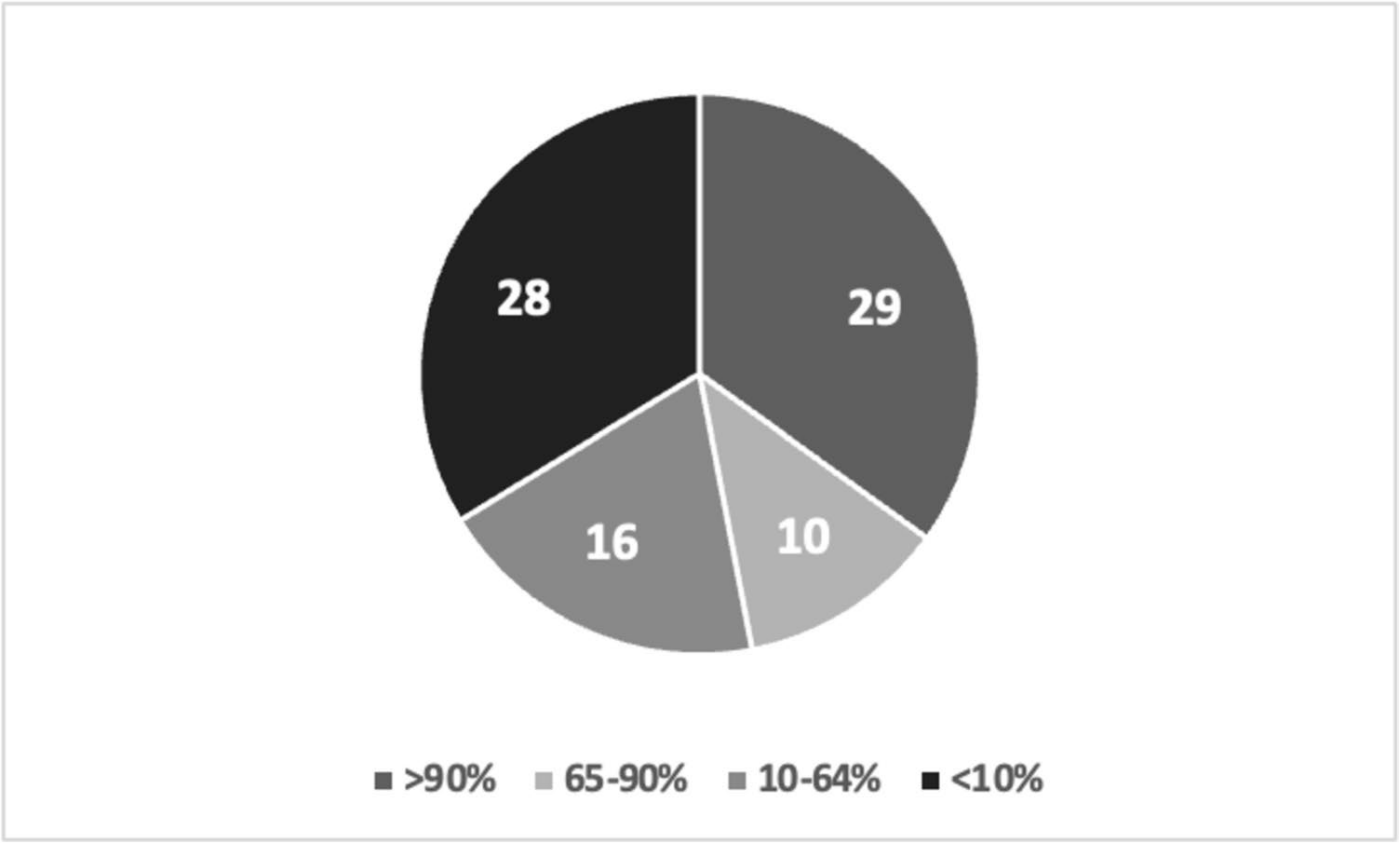
Safety glasses compliance according to the percent of the day used. Numbers indicate number of children belonging to each group

Eighty-two (99%) caregivers were aware of the recommendation given to their child to wear protective eyewear. Only one parent reported being unaware of this recommendation, even though it was documented in the child’s medical file. However, 26 (31%) caregivers did not know the true purpose of their child’s spectacles. Most commonly (*n* = 21/26, 81%) because they assumed glasses provided both protection and vision improvement, whereas they were prescribed for protection alone. Additionally, only eleven caregivers (13%) knew that the lens material used in their child’s glasses differed than what is typically used in regular children’s glasses. Seventy-five (90%) caregivers felt they received an adequate explanation from their treating physician about the importance of protective glasses for ocular trauma prevention and the implications it may have on their child’s life should it occur in the better-seeing eye. Nevertheless, regarding actual glasses usage, only 29 (35%) children wore safety glasses at least 90% of the day, 26 (31%) children used them part-time (10–90% of the day) and 28 (34%) used them seldom or never at all (< 10% of the day, Fig. 1).

Good compliance with wearing protective glasses (> 90% day) was not different among boys (14/44, 32%) compared with girls (15/39, 38%, *p* = 0.65), or when glasses were prescribed before three years of age (10/26, 38%) versus later in life (33%, 19/57, *p* = 0.80). However, as can be expected, compliance was higher when the glasses provided an optical correction. Fifty-six percent (14/25) of children wearing glasses with an optical correction wore them > 90% of the day versus 26% (15/58) of children wearing glasses for protective purposes alone (*p* = 0.01).

Thirty-three (40%) children participated in sports activities such as outdoor ball playing, martial arts practicing or horseback riding, in which protective eyewear is especially important. However, only 24 (73%) of them wore eye protection while practicing these activities.

Fifty-eight (70%) of children were older than six years of age at the time of surveying. Awareness of the importance of safety glasses and the advantage they offer was significantly different, with a higher value in the group of school-aged children (37/58, 64%) compared with younger children (7/25, 28%, *p* < 0.01); however, this increased awareness was not matched by increased glasses acceptance (18/58, 31% versus 11/25, 44%, *p* = 0.32).

**Fig. 2:**
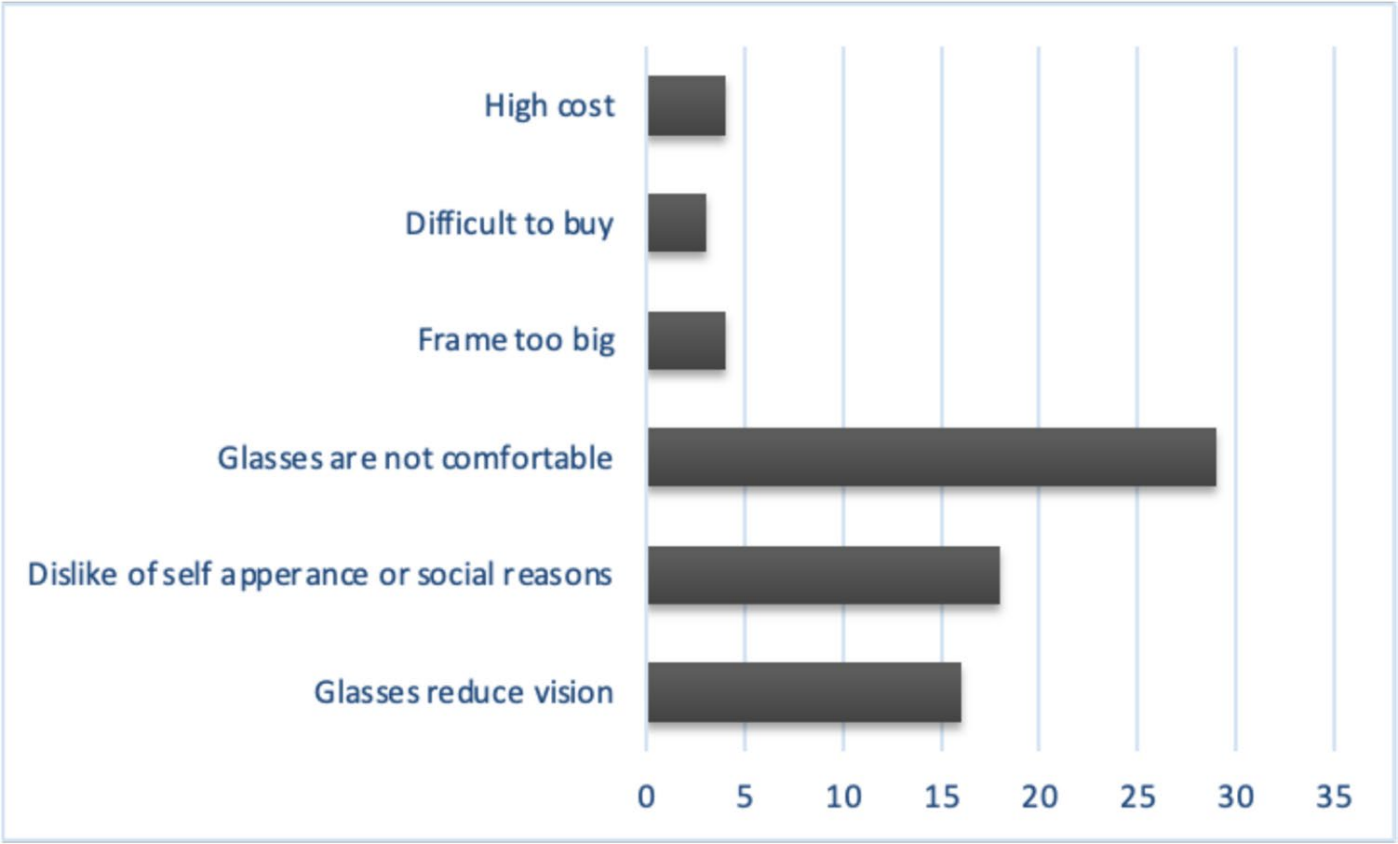
Reasons provided for non-compliance with protective eyewear

The three most common reasons provided by caregivers for safety glasses non-compliance were: discomfort (35%), dislike of self-appearance with glasses (22%), and lens induced vision reduction by dust, vapor, or lens scratches (16%). Additionally, three caregivers (4%) reported that they couldn't find protective eyewear in an optical shop, and four (5%) mentioned the glasses were too expensive, Fig. 2.

Twenty-three caregivers (27%) remembered at least one incident in which the safety glasses prevented an injury to the good eye, whereas five other caregivers (6%) recollected an injury event to the unprotected good eye while the child was not wearing protective eyewear. Two of these children (2%) began wearing safety glasses regularly afterwards. No ocular injuries occurred from the glasses themselves.

## Discussion

Eye protection is a proven method for preventing ocular trauma and blindness [1, 8, 9]. Eye safety precautions are especially important in one-eyed patients who are at increased risk of blindness from trauma to their good eye [5, 10], which may result in an indefinite change in lifestyle. Therefore, not recommending safety glasses to one-eyed patients may be regarded as suboptimal standard of care [6]. However, this recommendation cannot be enforced, and compliance is often limited [6, 7]. Ophthalmologists often cannot adequately predict which patient will follow their treatment recommendations. Prescribing glasses, including safety glasses in childhood, is no exception. Children in general often resist wearing glasses even when they offer a substantial optical correction [11, 12], let alone glasses having no refractive correction. There are conflicting data regarding safety glasses usage among one-eyed patients [6, 7, 13]. More than three decades ago, Drack et al. reported that 85% of enucleated children wore safety glasses more than half of their waking hours, including 33% who wore them constantly [6]. In another more recent study, rates of safety glasses usage among anophthalmic patients were much lower: two-thirds wore protective glasses either full-time (55%) or frequently (11%), whereas 29% never wore them and other 6% used them occasionally [7]. Our results show that non-anophthalmic children with reduced visual acuity in one eye used protective glasses even less often: only 35% wore eye protection at least 90% of the day, while 34% wore these glasses rarely or never at all, and the remainder wore them part-time only. Hence, we conclude that having functional monocular vision loss is associated with lower adherence with safety glasses compared with being truly one eyed, requiring a greater effort from physicians and caregivers to promote their use in this population.

Most cases of pediatric eye injuries occur during sports and recreational activities [2]. In the study by Drack et al. 100% of females and 80–93% of males wore safety glasses while performing sports activities [6]. In our study, only 73% of children wore eye protection during eye risk-sporting activities. We did not find a gender difference in wearing eye protection, although in the past, some researchers felt there was a stronger need for protective glasses in males because of gender-related differences in risks of ocular trauma [13].

Neimkin et al. found that patients wearing prescription glasses before undergoing enucleation and patients adherent to follow-up appointments were more likely to wear protective glasses afterward [7]. We similarly found that needing refractive correction for improving vision in the better-seeing eye increased protective glasses usage. The effect of age on wearing safety glasses is controversial. Drack et al. found that children beginning to wear them before three years of age are more likely to continue using them at an older age, possibly because the child becomes accustomed to wearing eye protection from an early childhood [6]. In our study we did not find such a correlation, similarly to the study reported by Neimkin et al. who also did not find an age effect [7].

Children often resist wearing spectacles needed to improve their vision due to multiple reasons, including general dislike to wearing glasses, peer pressure or teasing, or frame related facial discomfort [12]. Additional reported reasons for non-compliance relevant to wearing protective glasses include lack of interest or concern of potential vision loss, good uncorrected vision in the other eye and lenses induced disruption of vision [6, 7]. We found similar reasons for non-compliance. Interestingly, the current study did not find a correlation between awareness of safety glasses importance and their usage. However, we still think that educating caregivers and their children about the potential for eye injuries at home and during hazardous activities is an important first step to promoting eye protection. A possible cause for patients’ lack of interest and concern about eye trauma may be the media. Eye-safety measures including protective spectacles are seldom depicted in television scenes involving activities that are at high-risk for causing eye trauma and characters appearing in these scenes rarely suffer eye injuries despite not wearing proper eye protection [14, 15]. It is important to note that caregivers needed to purchase the recommended safety glasses, and some of them provided cost as the reason for non-compliance. Even though safety glasses are typically more expensive than regular glasses this added cost is negligible compared with the lifetime cost to society of pediatric blindness. Means should be employed to provide safety glasses to one-eyed children who cannot afford them. Frame appearance is another common deciding factor for glasses acceptance among children [11]. We similarly found that aesthetics was a major barrier to wearing safety glasses.

Recognizing common obstacles to safety glasses wear is an important step in devising measures to bridge them. In the current study, although nearly all caregivers surveyed were aware of the recommendation given to their child to wear eye protection and felt it was adequate, about a third of them did not understand its actual purpose. Hence, more accurate education may improve compliance. Other aspects that should be emphasized are the unique materials and construction used for protective glasses. Additionally, the risk involved in contact lens use in functionally one-eyed patients who require refractive error correction should also be explained. Contact lenses not only lack the desired protection provided by safety glasses but also harbor inherent health risks such as corneal infection [16]. Another aspect of patient counseling may be to tell patients about cases in which eye injury occurred while no protection was worn and other instances in which glasses prevented trauma. Several caregivers included in our study had this experience. Drack et al. also reported that 45% of their anophthalmic children remembered at least one incident in which safety glasses prevented an injury to the good eye [6].

This study has several limitations. It included several pediatric ophthalmologists from two medical centers making it impossible to verify the exact counseling each child and family received. Interview bias may be another limitation, as respondents may have reported higher than usual usage of safety glasses because they were embarrassed by not following the recommendations received, did not want to be criticized or because they overestimated their child’s true compliance. Additionally, because multiple interviewers conducted the telephone survey, it is possible that responses varied because interviewers might have emphasized different questions. Furthermore, caregivers of children with lower compliance might have been more reluctant to be interviewed for the same reasons. Finally, when questioned about eye trauma happening while their child was not wearing eye protection, respondents may have withheld information in case they felt that their negligence contributed to the accident.

In conclusion, compliance with safety glasses in functionally one-eyed children was lower than previously reported in anophthalmic children. Numerous gaps and barriers need to be bridged to improve eyewear protection in these children, to prevent trauma in the better-seeing eye with its devastating lifestyle effect.

## Appendix: Protective glasses telephone survey

- Does your child wear glasses?
- When did your child begin to wear glasses?
- Do you understand the function of the glasses? (protection/refraction/both)
- Are you aware of the material composition of the lenses?
- Are you aware of the frame’s material?
- Did you receive an explanation from the physician about the importance of safety glasses?
- What percentage of the day does your child wear the glasses?
  Over 90%, 65%-89%, 10%-64%, < 10% of waking hours.
- Does your child understand the importance of wearing protective glasses?
- Does your child have any objections to wearing safety glasses?
- Please provide the reasons for incompliance.
  Good vision without the glasses, glasses are not comfortable, appearance, lack of understanding or concern, cost.

Does your child participate in sports? If so which one?

Does your child wear protective glasses during sports?

Was you child injured in the eye while not wearing protective glasses?

Do you remember an event in which the protective glasses prevented eye injury?
